# Surveillance Outcomes of Respiratory Pathogen Infections During the 2021–2022 Season Among U.S. Military Health System Beneficiaries, October 3, 2021–October 1, 2022

**Published:** 2024-05-20

**Authors:** Bismark Kwaah, William E. Gruner, Laurie S. DeMarcus, Jeffrey W. Thervil, Whitney N. Jenkins, Fritz M. Castillo, Tamara R. Hartless, Victor K. Heh, Deanna Muehleman, Anthony C. Fries, Paul A. Sjoberg, Fabrice E. Evengue, Anthony S. Robbins

**Affiliations:** 1 U.S. Defense Health Agency Armed Forces Health Surveillance Division Air Force Satellite–U.S. Air Force School of Aerospace Medicine, Wright-Patterson Air Force Base, OH; 2 JYG Innovations, LLC, Dayton, OH; 3 Innovative Element, LLC, Beavercreek, OH; 4 U.S. Air Force School of Aerospace Medicine Epidemiology Laboratory, Wright-Patterson Air Force Base; 5 Department of Pathology and Area Laboratory Services, Landstuhl Regional Medical Center, Germany

## Abstract

The Department of Defense Global Respiratory Pathogen Surveillance Program conducts continuous surveillance for influenza, severe acute respiratory syndrome 2 (SARS-CoV-2), and other respiratory pathogens at 104 sentinel sites across the globe. These sites submitted 65,475 respiratory specimens for clinical diagnostic testing during the 2021-2022 surveillance season. The predominant influenza strain was influenza A(H3N2) (n=777), of which 99.9% of strains were in clade 3C.2a1b.2a2. A total of 21,466 SARS-CoV-2-positive specimens were identified, and 12,225 of the associated viruses were successfully sequenced. The Delta variant predominated at the start of the season, until December 2021, when Omicron became dominant. Most circulating SARS-CoV-2 viruses were subsequently held by Omicron sublineages BA.1, BA.2, and BA.5 during the season. Clinical manifestation, obtained through a self-reported questionnaire, found that cough, sinus congestion, and runny nose complaints were the most common symptoms presenting among all pathogens. Sentinel surveillance can provide useful epidemiological data to supplement other disease monitoring activities, and has become increasingly useful with increasing numbers of individuals utilizing COVID-19 rapid self-test kits and reductions in outpatient visits for routine respiratory testing.

**What are the new findings?:**

Department of Defense Global Respiratory Pathogen Surveillance Program data show that influenza A(H3N2) was the dominant subtype of influenza throughout the 2021-2022 surveillance season. Three coincident waves, 1 of influenza and 2 of SARS-CoV-2 activity, were observed during the season. The wave of influenza occurred in April 2022, while the SARS-CoV-2 waves occurred from January 2022 through April 2022 and again in July 2022.

**What is the impact on readiness and force health protection?:**

As the coronavirus disease (COVID-19) outbreak continues to evolve, it is crucial for health care providers and public health officials to be aware of the similarities as well as differences between SARS-CoV-2 (the causative agent of COVID-19), influenza, and other respiratory infections. These findings may contribute to improved clinical diagnoses and more effective management of respiratory infections among beneficiaries of the Military Health System.

## 1 BACKGROUND

In 1976, the U.S. Air Force Medical Service began conducting routine, global, laboratory-confirmed influenza surveillance. Efforts expanded when it became part of the Department of Defense Global Emerging Infections Surveillance and Response System (DOD-GEIS) in 1997.^1^ Since then, GEIS has provided central coordination and financial support for the Department of Defense Global Respiratory Pathogen Surveillance Program (DODGRPSP), which routinely collects respiratory specimens from U.S. Military Health System (MHS) beneficiaries who meet the COVID-19-like illness (CLI) or influenza-like illness (ILI) case definition or symptoms determined by a physician to be a CLI/ILI case (physician-diagnosed CLI/ILI).

Respiratory infections are common among U.S. military personnel, who often live in crowded conditions, work in stressful environments, and are frequently exposed to a variety of respiratory pathogens during deployments.^2^ It is crucial to conduct annual surveillance, to determine the circulating pathogens and detect changes for informing the DOD combatant commands’ critical decisions about force health protection. This report presents the incidence of respiratory pathogen infections and genetic characteristics of influenza, and severe acute respiratory syndrome-related coronavirus strain 2 (SARS-CoV-2) among MHS beneficiaries during the 2021-2022 surveillance season.

## 2 METHODS

DODGRPSP, a sentinel site-based program, requests that each site submit 6 to 10 specimens weekly with patient questionnaires from individuals who meet the CLI/ILI case definition or are physician-diagnosed CLI or ILI. Patient questionnaires are distributed with each collection kit and requested to be completed with each submitted specimen, but compliance is not always guaranteed. The CLI and ILI case definitions, respiratory specimen collection, and testing criteria, as well as other program information, have been previously described.^3,4,5^

Testing analyzed for this study was conducted in laboratories at Landstuhl Regional Medical Center (LRMC), Incirlik Medical Center, and the U.S. Air Force School of Aerospace Medicine (USAFSAM). Specimens positive for influenza or SARS-CoV-2 underwent genetic sequencing for further characterization, as previously described.5 Patients were classified by age group (children, 0-17 years; adults, 18-64 years, and elderly, 65+ years), geographic region (Eastern U.S., Western U.S., and outside continental U.S. [OCONUS]), and month of collection. Any specimens that the laboratory cancelled (52), rejected (347), did not test (795), or returned as an inconclusive test (141) were excluded. Individuals with multiple specimens (3,770) collected during the season were also removed from the study to avoid duplication, as they could have encountered several pathogens over the season.

All statistical analyses were performed using SAS version 9.4 (SAS Institute, Cary, NC). A *p*-value of <0.05 was considered statistically significant. Basic descriptive epidemiology was employed to obtain counts and rates of outcomes by sex, military beneficiary category, age group, month of collection, and geographic region. Patient symptoms among the 5 groups—influenza, other respiratory pathogens (adenovirus, seasonal coronavirus, human bocavirus, human metapneumovirus, and parainfluenza), respiratory syncytial virus (RSV), rhinovirus/enterovirus, and SARS-CoV-2—were performed using a chi-square or Fisher’s exact test, limited to those specimens associated with DODGRPSP questionnaires.

## 3 RESULTS

Between October 3, 2021 and October 1, 2022, a total of 65,475 respiratory specimens were tested, among which 26,794 (41%) specimens tested positive for respiratory pathogens (**Table 1**). About 61% of the specimens came from OCONUS, 22% were from the Western U.S., and 17% came from the Eastern U.S. SARS-CoV-2 (70.9%) and RSV (58.0%) were most detected at OCONUS sites, while influenza (45.0%) and rhinovirus/enterovirus (41.7%) were most detected in the Eastern U.S. Other pathogens (39.6%)—adenovirus, seasonal coronavirus, human bocavirus, human metapneumovirus, and parainfluenza—were detected more in the Western U.S. (**Table 1**).

**Figure 1:**
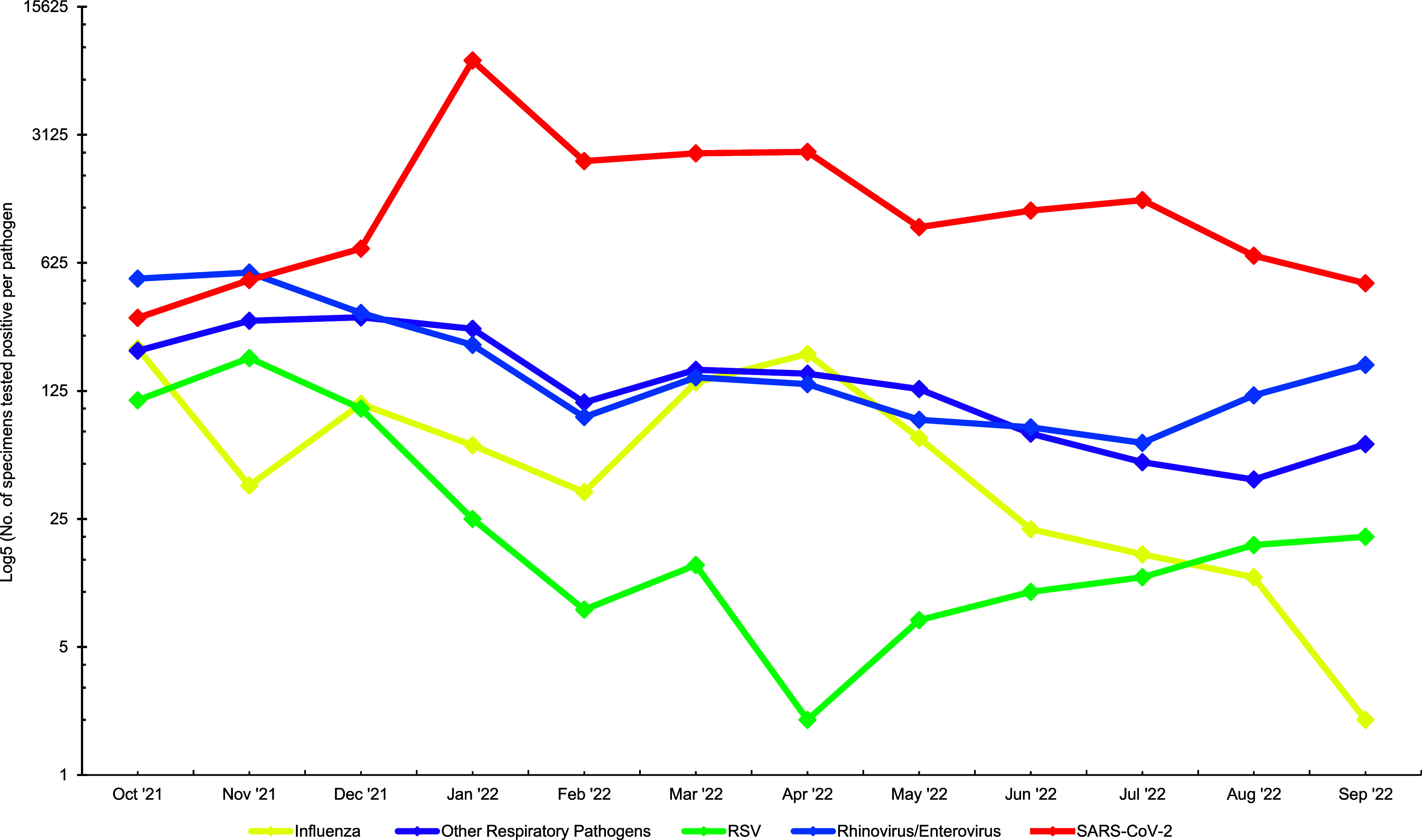
Numbers of Respiratory Pathogens that Tested Positive Among MHS Beneficiaries, October 2021–October 2022

**Figure 2:**
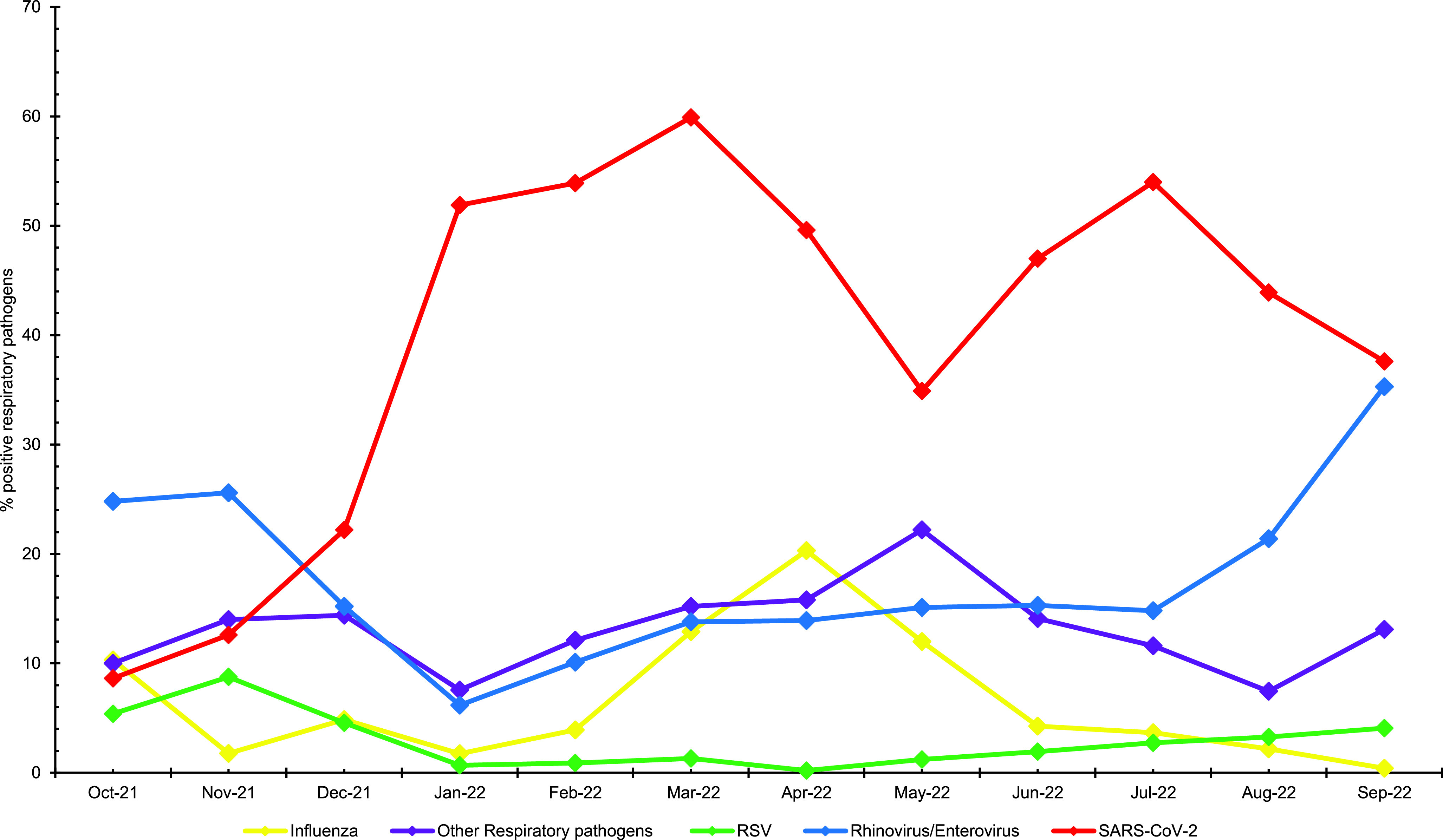
Percentages of Respiratory Pathogens that Tested Positive Among MHS Beneficiaries, October 2021–October 2022

Of the 65,475 specimens collected during the surveillance season, SARS-CoV-2 was detected in 32.8%, of which 8 were coinfections with influenza, including 4 influenza A(H3N2), 3 influenza A/not subtyped, 1 dual influenza and RSV (data not shown), and 65 were co-infections with other respiratory pathogens (**Table 2**). Rhinovirus/enterovirus (3.4%) was the second-most detected pathogen, followed by influenza (1.4%), seasonal coronavirus (0.9%), and RSV (0.6%). *Mycoplasma pneumoniae* and *Chlamydophila pneumoniae* were not detected during the season. The numbers of positive samples and positivity percentages, by specific pathogen and month of diagnosis, are shown in **Figures1** and **2**.

SARS-CoV-2 percent positivity increased to 52.0% in January 2022, then peaked at 60.0% in March 2022 (**Figure2**), corresponding to the predominance of Omicron BA.1 and BA.2 (**Figure3b**). Percent positivity decreased to as low as 35.0% during May 2022, then peaked again during early June 2022 (47.0%) through July 2022 (54.0%), before it decreased for the rest of the season (Figure 2). SARS-CoV-2 was the most prevalent pathogen detected during the season. In November 2021, however, the percent positivity of other respiratory pathogens as well as rhinovirus/enterovirus were briefly higher than SARS-CoV-2 (**Figure2**).

DODGRPSP data showed 1 distinct wave of influenza between mid-March to April 2022, with percent positivity peaking at 20.0% (**Figure2**). Among the 905 influenza viruses that were subtyped, influenza A(H3N2) was the predominant virus throughout the 2021-2022 surveillance season, which was in agreement with U.S. Centers for Disease Control and Prevention (CDC) data and the European Center for Disease Prevention and Control (ECDC).^6,7^ Most specimens testing positive for influenza A(H3N2) were detected in the Eastern U.S. (Health and Human Service regions 1, 2, 3).

The highest rates of influenza infections were observed among service members (72.0%), followed by children (15.8%). Positive influenza cases started relatively high (October 2021), then decreased until another positivity increase in March 2022, reaching the highest point in April 2022. This highest point was followed by a subsequent decrease around May 2022, through the end of the season. In contrast to the low October 2021 influenza activity demonstrated by the CDC and ECDC,^7,8^ the elevated influenza activity in DODGRPSP data was due to an influenza A(H3N2) outbreak at the U.S. Naval Academy.

Rhinovirus/enterovirus (26.0%) peaked in November 2021, then increased again between July 2022 (15.0%) and September 2022 (35.0%). Peak RSV (9.0%) activity was in November 2021 (**Figure2**), then declined until May 2022 (1.0%), when it steadily increased through September 2022 (4.0%). The highest percent positivity for RSV in participants was among children (56.8%), followed by service members (32.2%) (**Table 1**). Specimens grouped as other respiratory pathogens (22.0%) peaked in May 2022, but their activity and percent positivity remained steady throughout the season.

Symptomatic evaluation of patients was limited to those with a DODGRPSP questionnaire. Among the 65,475 specimens received and tested, 8,773 specimens also had DODGRPSP questionnaires, representing an approximate 13% questionnaire response rate. Questionnaires were not received from specimens tested at LRMC and Incirlik Medical Center during the season.

**Table 3** shows the distribution of demographic, clinical characteristics, and outcomes by viral agent. Chi-square tests were used to obtain *p*-values for the significance of the differences among the 5 groups. Significant associations were found between viral agent and gender, age group, and many symptoms. Males were more likely to be infected with influenza (69.3%) and SARS-COV-2 (68.0%) than with rhinovirus/enterovirus (58.6%), RSV (58.4%), or other respiratory pathogens (58.0%). Whereas the 0-17 year age group was more likely to be infected with RSV (57.6%), other pathogens (42.3%) or rhinovirus/enterovirus (34.0%) than influenza (14.6%) or SARS-COV-2 (11.6%). The 18-64 year age group was more likely to be infected with SARS-COV-2 (86.5%), influenza (85.4%) or rhinovirus/enterovirus (65.6%) than other respiratory pathogens (56.9%) or RSV (42.4%).

Cough (>80.0%), sinus congestion (>60.0%), and/or runny nose (>50.0%) were the most common presenting symptoms among all pathogens. Other frequent symptoms of patients with influenza, as well as SARS-CoV-2, were fatigue (>60.0%), headache (>70.0%), sore throat (>60.0%), body aches (>50.0%), and fever (>50.0%). Among participants positive for rhinovirus/enterovirus, RSV, or other pathogens, the most common symptoms were sore throat (>50.0%) and fatigue (>45.0%). The frequency of cough, sinus congestion, and runny nose symptoms among COVID-19 patients (>50.0%) was lower than in influenza patients (>60.0%); however, the frequency of patients with a loss of taste/smell (10.1%) was greater in patients with COVID-19 than in patients with any other pathogens (<8.0%) (**Table 3**). No significant associations were found between viral agents and diarrhea, acute respiratory distress, and shortness of breath (**Table 3**).

**Genetic Characteristics of Influenza and SARSCoV-2**

From October 1, 2021 through September 30, 2022, USAFSAM conducted next-generation sequencing and analysis on both influenza- and SARS-CoV-2-positive specimens. In total, 1,350 influenza sequences were either generated at USAFSAM or contributed by partner laboratories at the Navy Medical Research Unit 6 (NAMRU-6) in Peru or the Naval Health Research Center (NHRC) in San Diego, California. Ten influenza A(H1N1)pdm09 hemagglutinin (HA) sequences were characterized, of which 2 were clade 6B.1A.5a1 and 8 were clade 6B.1A.5a2. Of the 1,339 influenza A(H3N2) HA sequences characterized, 1 was clade 3C.2a2b.1a and the remaining 1,338 were clade 3C.2a1b.2a2 (subgrouping shown in **Figure3a**). The predominant influenza strain of the season was A(H3N2), of which 99.9% of strains were clade 3C.2a1b.2a2. The subgroup sharing D53G held the majority for most of the season, although at times the subgroup sharing D53N was in the majority. By the end of the season, the subgroup sharing E50K became the dominant group.

One influenza B/Yamagata HA sequence was characterized as clade Y3, however the possibility of this being a live, attenuated influenza vaccine (LAIV) strain has not been eliminated. In addition, 12,225 out of 21,466 SARS-CoV-2-positive specimens were sequenced, and 10,381 were assigned to PANGO lineages. Among those lineages, 1 was an Alpha variant, 1,864 were Delta variants, 8,510 were Omicron variants, and 6 were recombinant viruses. The Omicron variants were divided into sublineages: 3,794 BA.1; 2,572 BA.2 including 622 BA.2.12.1; 18 BA.3; 324 BA.4 including 65 BA.4.6; and 1,802 BA.5 (**Figure3b**).

## 4 DISCUSSION

The DODGRPSP data, along with the U.S. general population, saw the return of influenza after being relatively absent in the previous season.^5,6^ The overall results revealed a positivity rate of 41.0% among all viruses; SARS-CoV-2 remained prevalent, however, and continued to be the dominant virus circulating among MHS beneficiaries. These data also show that rhinovirus/enterovirus was the second-most dominant virus in circulation, which increased in positivity starting in July 2022.

The overwhelming majority of clade 3C.2a1b.2a2, also reflected in data from the CDC, prompted the selection of A/Darwin/9/2021-like virus for the egg-propagated strain and A/Darwin/6/2021-like virus for the cell- and recombinant-based strain of the 2022-2023 influenza vaccine A(H3N2) component.^9^ Although this clade persisted throughout the season, several subgroups emerged that could have potentially altered vaccine strain efficacy. Following the 2021-2022 season, the subgroup sharing D53G was renamed clade 2a.1 with associated subclades, the subgroup sharing D53N was renamed clade 2a.3 with associated subclades, the subgroup sharing E50K was renamed 2b with associated subclades, and the subgroup sharing 205F was renamed clade 2c with associated subclades. No change was made to the influenza A(H1N1)pdm09 or influenza B/Yamagata vaccine component. While no influenza B/Victoria specimens were sequenced by USAFSAM, the vaccine component was changed for the 2022-2023 season due to global circulation of some diversified strains.

The 2021-2022 season started with almost entirely Delta variants of SARS-CoV-2 until December 2021, when Omicron emerged and became dominant. Sublineages BA.1, BA.2, and BA.5 then subsequently held most circulating SARS-CoV-2 viruses for the rest of the season. The positivity rates showed 2 distinctive peaks, 1 in January 2022 through March 2022 (coinciding with Omicron sub-lineage BA.1/BA.2) and 1 in July 2022 (coinciding with Omicron sub-lineage BA.5) (**Figures2** and **3b**), which qualitatively agree with previous reports on the positivity rate of Omicron worldwide.^10,11^

The end of September 2022 showed a reduction in the overall positivity rate. It should be noted that almost all detected influenza lineages, as well as SARS-CoV-2 variants and subvariants, were found in all geographic regions, suggesting that newly introduced viral strains can spread to all regions.

In this study, the SARS-CoV-2 infection rate in the 0-17 year age group was lower compared to any other pathogen, while RSV cases were predominantly among 0-17 year-olds. In contrast, SARS-CoV-2 was the most frequent virus detected among adults (18-64) (**Table 3**). The findings of this report are consistent with other studies concerning the impact of SARS-CoV-2 among adults and RSV on children.^12,13,14^

This study had some limitations: First, the division of viral agents into only 5 groups, including 1 group representing 5 different pathogens, may be associated with different symptoms. Linking the other respiratory pathogens group as one group is due to small sample sizes, and this can only be possible when symptoms of the combined pathogens are similar. For instance, studies have shown that fever was not associated with adenovirus and parainfluenza virus.^15^ The study also reveals that general symptoms such as cough, sinus congestion, and sore throat are more likely to be found in patients with other respiratory pathogens, of which it cannot be ascertained since it involves 5 different pathogens.

Secondly, DODGRPSP questionnaires had a low response rate, of about 13%. Even when statistically significant, symptomology results must be interpreted with caution, as a large volume of specimens were submitted without a questionnaire. All specimens met the CLI/ILI case definition, however, or specimens were determined by a physician to be a CLI/ILI case.

During the 2021-2022 surveillance season, the temporal pattern of SARS-CoV-2 and influenza positivity among MHS beneficiaries was largely consistent with overall U.S. SARS-CoV-2 and influenza surveillance data, supporting the proposition that sentinel surveillance provides an accurate representation of respiratory pathogens trends.^6,16,17^ These results emphasize the need for continuous surveillance of multiple respiratory pathogens and identification of novel pathogens, along with use of a CLI/ILI case definition for effective public health management and force health protection. Sentinel surveillance remains crucial for detecting emerging strains and guiding vaccine development efforts.

## Figures and Tables

**Table 1:** Characteristics of Surveillance Population and Specimen Sources, MHS Beneficiaries, 2021-2022 Surveillance Season

	SARS-CoV-2		Influenza		Rhino/Entero		RSV		ORP^a^		No Pathogen Detected^b^		Negative^c^		Total	
	No.	%	No.	%	No.	%	No.	%	No.	%	No.	%	No.	%	No.	%
Total	21,466	32.8	913	1.4	2,226	3.4	419	0.6	1,770	2.7	9,723	14.8	28,958	44.2	65,475	100.0
Sex																
Male	13,050	60.8	634	69.4	1,360	61.1	235	56.1	1,033	58.4	5,953	61.2	18,333	63.3	40,598	62.0
Female	8,416	39.2	279	30.6	866	38.9	184	43.9	737	41.6	3,770	38.8	10,625	36.7	24,877	38.0
Age group, y																
0-17	4,415	20.5	148	16.2	645	29.0	238	56.8	730	41.2	1,502	15.4	5,183	17.9	12,861	19.6
18-64	16,842	78.5	763	83.6	1,570	70.5	180	43.0	1,025	58.0	8,078	83.1	23,336	80.6	51,794	79.1
65+	209	1.0	2	0.2	11	0.5	1	0.2	15	0.8	143	1.5	439	1.5	820	1.3
Month of collection																
October	313	1.5	213	23.3	459	20.6	81	19.3	208	11.8	1,022	10.5	3,314	11.4	5,610	8.6
November	503	2.3	38	4.2	493	22.1	157	37.5	293	16.6	1,077	11.1	3,477	12.0	6,038	9.2
December	748	3.5	106	11.6	292	13.1	85	20.3	295	16.7	1,332	13.7	2,617	9.0	5,475	8.4
January	7,941	36.9	62	6.8	196	8.8	19	4.5	250	14.1	3,032	31.2	7,376	25.5	18,876	28.8
February	2,247	10.5	35	3.8	76	3.4	7	1.7	101	5.7	654	6.7	1,922	6.6	5,042	7.7
March	2,472	11.5	139	15.2	133	6.0	11	2.6	158	8.9	611	6.3	1,653	5.7	5,177	7.9
April	2,518	11.7	199	21.8	118	5.3	1	0.2	138	7.8	488	5.0	2,563	8.9	6,025	9.2
May	979	4.6	69	7.6	68	3.1	7	1.7	116	6.6	286	2.9	1,828	6.3	3,353	5.1
June	1,205	5.6	22	2.4	72	3.2	9	2.1	64	3.6	334	3.4	1,359	4.7	3,065	4.7
July	1,375	6.4	16	1.8	56	2.5	9	2.1	45	2.5	294	3.0	1,173	4.1	2,968	4.5
August	682	3.2	12	1.3	109	4.9	16	3.8	41	2.3	362	3.7	874	3.0	2,096	3.2
September	483	2.3	2	0.2	154	6.9	17	4.1	61	3.4	231	2.4	802	2.8	1,750	2.7
Geographic region^d^																
Eastern U.S.	2,447	11.4	411	45.0	928	41.7	73	17.4	597	33.7	3,669	37.7	2,985	10.3	11,110	17.0
Western U.S.	3,792	17.7	158	17.3	742	33.3	103	24.6	701	39.6	3,129	32.2	5,725	19.8	14,350	21.9
Outside continental U.S.	15,227	70.9	344	37.7	556	25.0	243	58.0	472	26.7	2,925	30.1	20,248	69.9	40,015	61.1
Beneficiary category																
Adult	5,520	25.7	110	12.0	294	13.2	45	10.7	214	12.1	1,829	18.8	6,098	21.1	14,110	21.5
Child	4,415	20.6	144	15.8	644	28.9	238	56.8	729	41.2	1,498	15.4	5,183	17.9	12,851	19.6
Elderly	209	1.0	2	0.2	11	0.5	1	0.2	15	0.8	143	1.5	439	1.5	820	1.3
Service member	11,322	52.7	657	72.0	1,277	57.4	135	32.2	812	45.9	6,253	64.3	17,238	59.5	37,694	57.6
Data source																
INCIRLIK	547	2.5	6	0.6	30	1.3	5	1.2	25	1.4	96	1.0	1,095	3.8	1,804	2.8
LRMC	14,558	67.8	165	18.1	310	13.9	196	46.8	287	16.2	2,004	20.6	19,152	66.1	36,672	56.0
USAFSAM	6,361	29.6	742	81.3	1,886	84.7	218	52.0	1,458	82.4	7,623	78.4	8,711	30.1	26,999	41.2

Abbreviations: MHS, Military Health System; SARS-CoV-2, severe acute respiratory syndrome-related coronavirus strain 2; Rhino/Entero, rhinovirus/enterovirus; RSV, respiratory syncytial virus; ORP, other respiratory pathogen; USAFSAM, U.S. Air Force School of Aerospace Medicine; LRMC, Landstuhl Regional Medical Center.

^a^Adenovirus, coronavirus, human bocavirus, human metapneumovirus, parainfluenza.

^b^No pathogen was identified via multiplex testing and may not include SARS-CoV-2 testing.

^c^Specimen was negative for SARS-CoV-2 and only tested for SARS-CoV-2.

^d^Eastern U.S. includes regions 1-5; Western U.S. includes regions 6-10; regions 1-10 are U.S. Health and Human Services Regions.

**Table 2:** SARS-CoV-2, Influenza, and Other Respiratory Pathogens Among MHS Beneficiaries, 2021–2022 Surveillance Season

Pathogen	No. of specimens	Total (%)
Total	65,475	100
**SARS-CoV-2 detected**	21,466	32.8
Single infection	21,401	99.7
Co-infection with non-influenza respiratory pathogen	65	0.3
Co-infection with influenza	8	<0.01
**Influenza detected**	905	1.4
A(H1N1)pdm09	8	0.9
A(H3N2)	777	85.9
A / not subtyped	119	13.1
**Other respiratory pathogen**	4,415	6.7
Adenovirus	86	1.9
Coronavirus (seasonal)	573	13.0
Human bocavirus	66	1.5
Human metapneumovirus	348	7.9
Parainfluenza	357	8.1
Respiratory syncytial virus (RSV)	419	9.5
Rhinovirus/enterovirus	2,226	50.4
Non-influenza viral coinfection	340	7.7
**Other**	38,681	59.1
No pathogen detected^a^	9,723	25.1
Negative^b^	28,958	74.9

Abbreviations: SARS-CoV-2, severe acute respiratory syndrome-related coronavirus strain 2; MHS, Military Health System; No., number.

^a^No pathogen was identified via multiplex testing.

^b^Specimen was negative for SARS-CoV-2 and only tested for SARS-CoV-2.

**Figure 3a:**
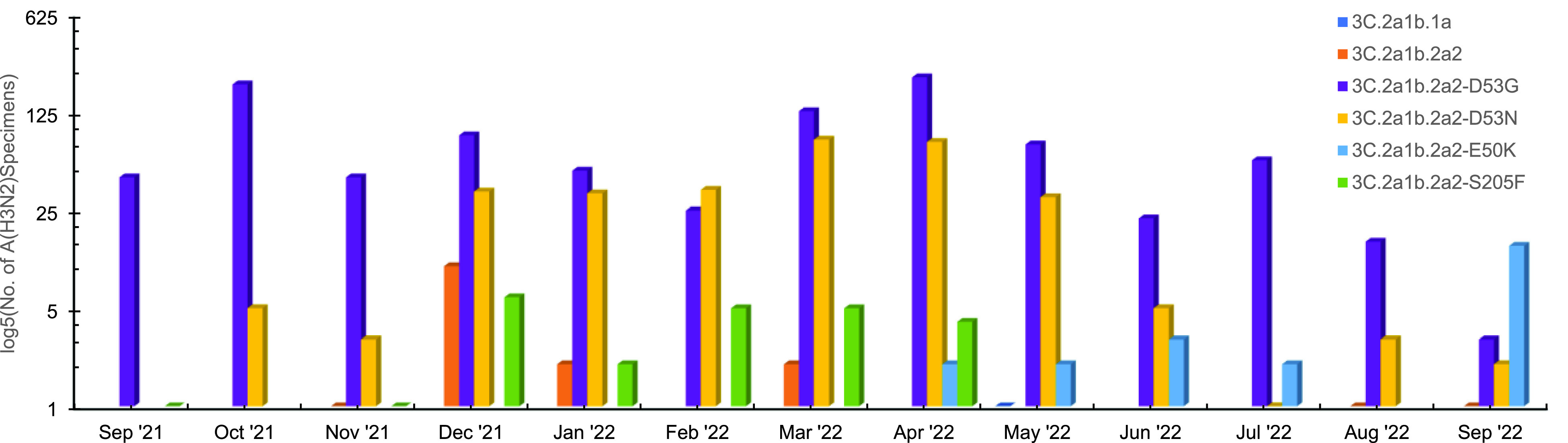
Influenza A(H3N2) Clade Proportions Among MHS Beneficiaries, 2021–2022 Surveillance Season (n=1,339)

**Figure 3b:**
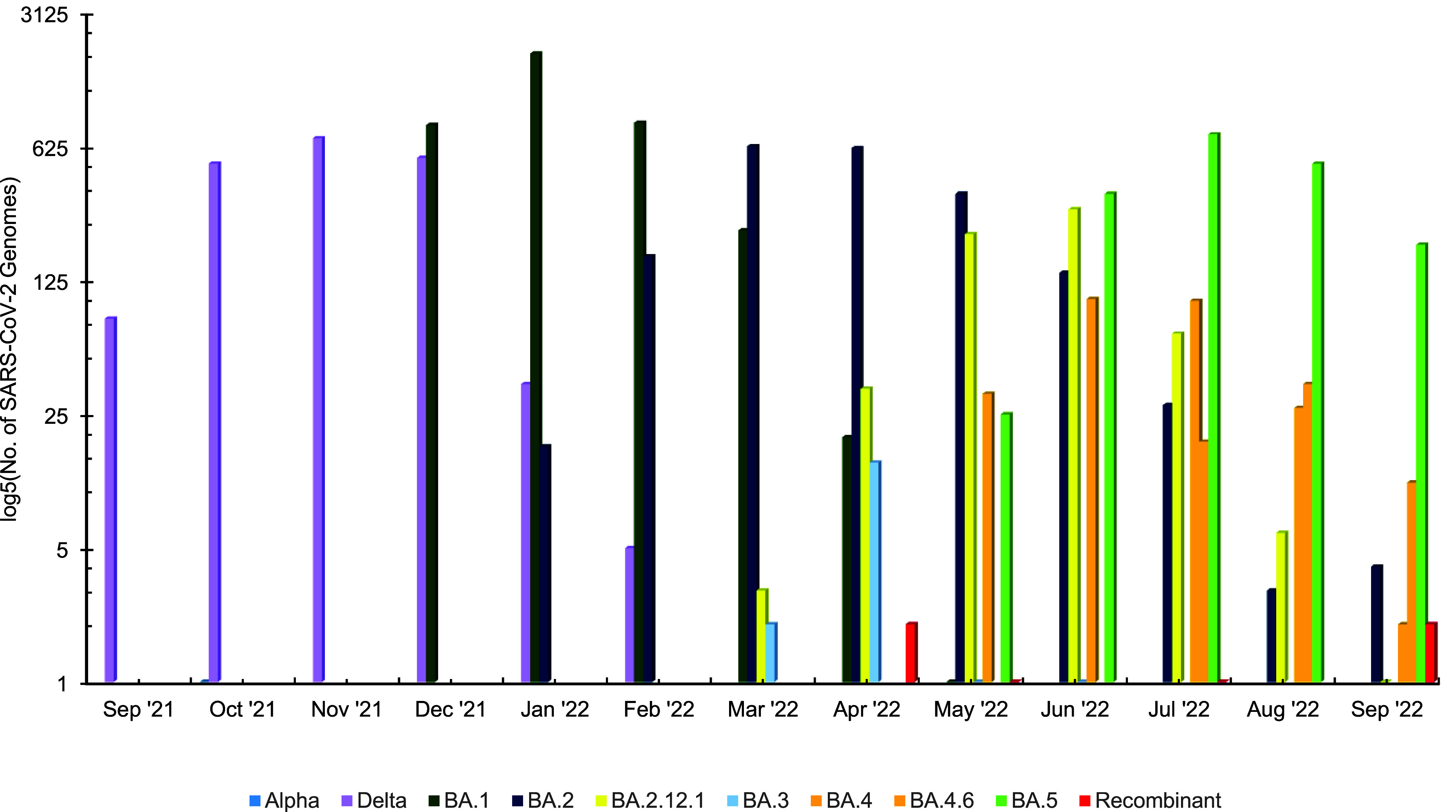
SARS-CoV-2 Lineages Identified Among MHS Beneficiaries, 2021–2022 Surveillance Season (n=12,225)

**Table 3:** Demographic and Clinical Details of MHS Beneficiaries, by Viral Agent, 2020–2021 Surveillance Season

Variable	SARS-CoV-2		Flu		Rhino/Entero		RSV		ORP^a^		p-value
	n=749		n=570		n=1,629		n=243		n=1,161		
	No.	%	No.	%	No.	%	No.	%	No.	%	
**Sex**											<.001
Male	509	68.0	395	69.3	954	58.6	142	58.4	673	58.0	
Female	240	32.0	175	30.7	675	41.4	101	41.6	488	42.0	
**Age group, y**											<.001
0-17	87	11.6	83	14.6	555	34.0	140	57.6	491	42.3	
18-64	648	86.5	487	85.4	1,068	65.6	103	42.4	661	56.9	
65+	14	1.9	0	0.0	6	0.4	0	0.0	9	0.8	
**Symptom**											
Cough	562	79.9	502	91.4	1,234	79.4	213	90.3	919	82.7	<.001
Sore throat	471	69.0	385	73.9	953	64.9	107	51.4	579	57.0	<.001
Fatigue	407	61.3	388	75.9	798	54.9	101	47.9	508	50.4	<.001
Body aches	400	58.8	378	72.0	534	37.5	36	18.3	366	36.9	<.001
Chills	319	48.3	345	66.1	438	31.0	40	20.1	309	31.0	<.001
Headache	472	69.5	386	74.1	788	54.8	59	29.5	476	48.0	<.001
Runny nose	329	49.9	342	67.9	1,093	73.0	166	75.8	753	71.2	<.001
Sinus congestion	467	69.1	389	76.3	1,249	81.3	183	82.8	868	80.4	<.001
Fever	384	57.6	378	72.6	643	44.7	125	57.9	581	55.3	<.001
Shaking	104	16.7	115	24.2	108	7.9	4	2.0	68	7.1	<.001
Vomit	43	7.0	59	12.2	160	11.6	22	10.7	93	9.7	0.016
Taste/smell	62	10.1	23	4.9	100	7.4	15	7.7	58	6.2	0.014
Diarrhea	65	10.4	63	13.2	169	12.2	12	6.1	118	12.5	0.062
Acute respiratory distress	13	2.5	15	4.0	35	3.0	3	1.9	24	3.0	0.635
Shortness of breath	112	17.8	80	16.6	247	17.9	34	17.1	160	16.7	0.939

Abbreviations: MHS, Military Health System; SARS-CoV-2, severe acute respiratory syndrome-related coronavirus strain 2; Flu, influenza; Rhino/Entero, rhinovirus/enterovirus; RSV, respiratory syncytial virus; No., number; N, Number; ORP, other respiratory pathogen; y, years.

^a^Adenovirus, coronavirus, human bocavirus, human metapneumovirus, parainfluenza.

## AUTHOR AFFILIATIONS

U.S. Defense Health Agency Armed Forces Health Surveillance Division Air Force Satellite–U.S. Air Force School of Aerospace Medicine, Wright-Patterson Air Force Base, OH: Mr. Kwaah, Ms. DeMarcus, Mr. Thervil, Ms. Jenkins, Ms. Hartless, Dr. Heh, Dr. Sjoberg, Dr. Evengue, Lt Col Robbins; JYG Innovations, LLC, Dayton, OH: Mr. Gruner, Dr. Muehleman; Innovative Element, LLC, Beavercreek, OH: Mr. Kwaah, Ms. DeMarcus, Mr. Thervil, Ms. Jenkins, Ms. Hartless, Dr. Sjoberg, Dr. Heh; U.S. Air Force School of Aerospace Medicine Epidemiology Laboratory, Wright-Patterson Air Force Base: Mr. Gruner, Dr. Fries, Dr. Muehleman; Department of Pathology and Area Laboratory Services, Landstuhl Regional Medical Center, Germany: Mr. Castillo
